# Supplementary material for: Scoping review of qualitative studies on family planning in Uganda
Source: PLOS Glob Public Health. 2024 Jul 3;4(7):e0003313. doi: 10.1371/journal.pgph.0003313 (PMC11221757; doi:10.1371/journal.pgph.0003313)
Supplement: Attachment — Submitted filename: FP Review Uganda Response to Reviewers.docx [file pgph.0003313.s002.docx]

9 March 2024

PLOS Global Public Health Editorial Board

**Title: "Scoping review of qualitative studies on family planning in Uganda” (PGPH-D-23-02496)**

Dear Marianne Clemence, Staff Editor, PLOS Global Public Health:

We are very pleased to have the opportunity to revise our original manuscript for publication in *PLOS Global Public Health*. We addressed each of the reviewer’s comments and made the necessary revisions and modifications. Responses are provided below.

**Journal Requirements:**

When submitting your revision, we need you to address these additional requirements.

**Requirement #1:** We noticed you have some minor occurrence of overlapping text with the following previous publication(s), which needs to be addressed:

- <https://doi.org/10.1155/2023/7583550>
-<https://doi.org/10.1186/1742-4755-11-1>

In your revision ensure you cite all your sources (including your own works), and quote or rephrase any duplicated text outside the methods section. Further consideration is dependent on these concerns being addressed.

**Response #1:** Thank you, we revised the overlapping text for the previous publication <https://doi.org/10.1186/1742-4755-11-1> citation. We are unable to find the occurrence of overlapping text with the previous publication <https://doi.org/10.1155/2023/7583550>. If the journal could provide the instances of duplicated text outside the methods section we would be happy to update the manuscript. We ensured that we cited all our sources outside the methods section.

**Staff Editor Comments:**

**Comment #1:** The manuscript has been evaluated by two reviewers, and their comments are available below. Reviewer #1 has raised concerns about the theoretical framework underpinning your analysis and the interpretation of your findings, whilst Reviewer #2 requests revisions to the Introduction and risk of bias assessments.

Risk of bias assessments are not typically included as part of a scoping review. However, as part of your revisions could you please clarify why you consider that your research question is more suited to a scoping review rather than a systematic review. Although Reviewer #1 has requested more discussion about how results observed in these articles might affect FP and CAC programming, please ensure that your scoping review does not speculate on implications for practice or clinically relevant questions.

**Response #1:** We clarified why we consider that our research question is more suited to a scoping review rather than a systematic review in response to the reviewer comment. We ensured that our scoping review does not speculate on implications for practice or clinically relevant questions.

**Reviewer comments:**

**Reviewer #1**

**Comment #1:** The topic of drivers of family planning and CAC choices is an important one, and this article is systematically researched and analyzed.

**Response #1:** Thank you!

**Comment #2:** The theory of planned behavior as an analytical framework underemphasizes the enormous effect of provider influence, structural gender norms, service quality, policy and policy implementation, and systemic barriers to women's mobility and financial means. While a study of individual level influences on FP- and CAC-related choices may lend value it should be analyzed using a framework that gives greater emphasis to the many factors at the health system, sturcural, and political levels. I recommend using the same results but reanalyzing using a more holistic theoretical framework.

**Response #2:** Thank you for sharing this observation. We followed your recommendation and used the same results but reanalyzed using the more holistic *Ecological Systems Theory*. We replaced the figure highlighting the *Theory of Planned Behavior* with one presenting a comprehensive overview of the *Ecological Systems Theory* for FP in Uganda.

**Comment #3:** While the discussion was thorough, the application of the results to programing, service provision, or policy was not robust. The article needs a thoughtful discussion of how these results observed in these articles might affect FP and CAC programming.

**Response #3:** We appreciate this suggestion for improvement and revised the text providing a thoughtful discussion of how results observed in these articles might affect FP and CAC programming.

**Reviewer #2**

**Comment #1:** This review focuses on an interesting and important topic around FP in Uganda. The search strategy is sound and the findings have been synthesised well using the TPB constructs to guide themes.

**Response #1:** Thank you!

**Comment #2:** The introduction would benefit from providing further data and evidence highlighting the prevalence of unintended pregnancy and high risk pregnancy recorded in Uganda. This will show the problem clearer and justify why looking to improve FP in Uganda is required.

**Response #2:** We appreciate this suggestion for improvement and expanded the introduction section to provide further data and evidence highlighting the annual rates of unintended pregnancies and the proportion of them ending in abortion in Uganda.

**Comment #3:** The Methods should include a risk of bias assessment of each article included to ensure added rigour in justifying which studies were included. Using JBI critical appraisal tools for example https://jbi.global/critical-appraisal-tools can be used to assess bias across various types of articles. This is just one resource that can be used. The same two independent reviewers can undertake these assessments and include the risk of bias table as a supplementary file.

**Response #3:** As the Staff Editor mentioned in their comment, risk of bias assessments are not typically included as part of a scoping review. We state in our introduction (lines 67-68) our research question aims to identify "qualitative studies aims to provide an overview of the current state of knowledge, attitudes, and practices of family planning and comprehensive abortion care in Uganda." This aim is in line with the goals of a scoping review as discussed below.

When one is mapping and exploring the literature on a topic, a scoping review is most appropriate. One is exploring, providing an overview of the literature. A scoping review allows for less restrictive inclusion criteria whereas the research question addressed for in systematic review is more specific in nature.

In general, the research question/query for a scoping review has a broader scope and fulfills the PCC question format (Population, Concept, Query). A systematic review question is more precise in nature and can often be defined using the PICO question model (Population, Intervention, Comparator, and Outcome).

A scoping review aids in exploring the existing literature on a broader query (additionally, sub-questions can be explored). A systematic review critically appraises the literature on a more precise question to answer (or come to a conclusion) on a more specific question. In some cases, a scoping review is used as a precursor (after "scoping" the literature) to a systematic review.

References:

Munn, Z., Peters, M.D.J., Stern, C. et al. Systematic review or scoping review? Guidance for authors when choosing between a systematic or scoping review approach. BMC Med Res Methodol 18, 143 (2018). https://doi.org/10.1186/s12874-018-0611-x

11.1.2 Scoping reviews compared to other types of review—JBI Manual for Evidence Synthesis—JBI Global Wiki. (n.d.). Retrieved February 5, 2024, from <https://jbi-global-wiki.refined.site/space/MANUAL/4687789/11.1.2+Scoping+reviews+compared+to+other+types+of+review>

We thank you for your consideration of this manuscript for publication and look forward to hearing from you.

Sincerely,

The Authors
